# Complete Genome of *Bacillus thuringiensis* Myophage BigBertha

**DOI:** 10.1128/genomeA.00853-13

**Published:** 2013-12-05

**Authors:** Jose H. Ting, Trinity B. Smyth, Karthik R. Chamakura, Gabriel F. Kuty Everett

**Affiliations:** Center for Phage Technology, Texas A&M University, College Station, Texas, USAa; Department of Biology, University of Mary Washington, Fredericksburg, Virginia, USAb

## Abstract

BigBertha is a myophage of *Bacillus thuringiensis*, a widely used biocontrol agent that is active against many insect pests of plants. Here, we present the complete annotated genome of BigBertha. The genome shares 85.9% sequence identity with *Bacillus cereus* phage B4.

## GENOME ANNOUNCEMENT

*Bacillus thuringiensis* is a Gram-positive soil-dwelling bacterium. During sporulation, *B. thuringiensis* produces exotoxins that are deadly when ingested by insects (1). As a result, *B. thuringiensis* is commonly used as an environmentally friendly insecticide (2). Here, we describe the genome of the *B. thuringiensis* myophage BigBertha*.*

BigBertha was isolated from a soil sample collected in Fredericksburg, VA. Phage DNA was sequenced using 454 pyrosequencing at the Emory GRA Genome Center (Emory University, Atlanta, GA). Trimmed FLX Titanium reads were assembled to a single contig at 145.6-fold coverage using the Newbler assembler version 2.5.3 (454 Life Sciences) with the default settings. The contig was confirmed to be complete by PCR. Genes were predicted using GeneMarkS (3) and corrected using software tools available on the Center for Phage Technology (CPT) portal (https://cpt.tamu.edu/cpt-software/portal/). Transmission electron microscopy was performed at the University of Mary Washington.

The unique genome of BigBertha is 162.6 kb, with a coding density of 92.3% and a G+C content of 37.8%. The TerL of BigBertha is homologous to the TerLs of phages with long terminal repeats. Processing the raw sequencing data with the Pause (https://cpt.tamu.edu/cpt-software/releases/pause/) method showed the terminal redundancy to be 2,577 bp. A total of 287 genes were identified in the unit genome, among which 32 are novel hypothetical, 202 are conserved hypothetical, and 53 had BLAST hits that matched known proteins. BigBertha shares homology with *Bacillus cereus* bacteriophage B4, with 219 of the 287 genes being comparable and ordered in a similar organization. BigBertha also infects avirulent *Bacillus anthracis* (delta Sterne).

BigBertha contains genes that code for proteins involved in DNA replication and recombination, including DNA polymerase, helicase, primase, RecA, Holliday junction resolvase, DNA binding proteins, and three recombination nucleases. Genes related to DNA and amino acid biosynthesis were found (*S*-adenosyl methionine [SAM] methyltransferase, ribonucleotide reductase subunits alpha and beta, dUTPase, dihydrofolate reductase, and thymidylate synthase). BigBertha also encodes two sigma factors and three transcriptional regulators.

The structural genes identified include those encoding a portal protein, prohead protease, major capsid protein, tail sheath protein, tail tube protein, a tailspike with a pectin lyase domain, two tail lysins, tail fiber, and baseplate assembly proteins (P2 W and J). The major capsid protein is related to the major capsid protein of *Staphylococcus* phages K and G1. Interestingly, the major capsid protein has a predicted C-terminal transmembrane domain. How and if this is involved in phage assembly remain to be determined. The lysis genes identified encode a class II holin with two transmembrane domains in an N-in C-in topology and an l-alanyl-d-glutamate peptidase.

BigBertha also encodes an Ftsk/SpoIIIE protein. The SpoIIIE DNA translocase plays a vital role in sporulation. It is required to translocate the chromosomal DNA into the forespore from the mother cell during the polar septation event (4). It is also needed for engulfment of the forespore (5, 6). The implications of the presence of a SpoIIIE homologue in BigBertha are currently unknown.

### Nucleotide sequence accession number.

The genome sequence of phage BigBertha was contributed as accession no. KF669647 to GenBank.
